# CD200R activation on naïve T cells by B cells induces suppressive activity of T cells via IL-24

**DOI:** 10.1007/s00018-024-05268-2

**Published:** 2024-05-23

**Authors:** Kuan-Hua Chu, Bor-Luen Chiang

**Affiliations:** 1 Department of Pediatrics, National Taiwan University Hospital, Taipei, Taiwan; 2 Genome and Systems Biology Degree Program, College of Life Science, National Taiwan University, Taipei, Taiwan; 3 Allergy Center, National Taiwan University Hospital, Taipei, Taiwan

**Keywords:** CD200, IL-24, Treg cell, Peyer’s patch, B cells

## Abstract

CD200 is an anti-inflammatory protein that facilitates signal transduction through its receptor, CD200R, in cells, resulting in immune response suppression. This includes reducing M1-like macrophages, enhancing M2-like macrophages, inhibiting NK cell cytotoxicity, and downregulating CTL responses. Activation of CD200R has been found to modulate dendritic cells, leading to the induction or enhancement of Treg cells expressing Foxp3. However, the precise mechanisms behind this process are still unclear. Our previous study demonstrated that B cells in Peyer’s patches can induce Treg cells, so-called Treg-of-B (P) cells, through STAT6 phosphorylation. This study aimed to investigate the role of CD200 in Treg-of-B (P) cell generation. To clarify the mechanisms, we used wild-type, STAT6 deficient, and IL-24 deficient T cells to generate Treg-of-B (P) cells, and antagonist antibodies (anti-CD200 and anti-IL-20RB), an agonist anti-CD200R antibody, CD39 inhibitors (ARL67156 and POM-1), a STAT6 inhibitor (AS1517499), and soluble IL-20RB were also applied. Our findings revealed that Peyer’s patch B cells expressed CD200 to activate the CD200R on T cells and initiate the process of Treg-of-B (P) cells generation. CD200 and CD200R interaction triggers the phosphorylation of STAT6, which regulated the expression of CD200R, CD39, and IL-24 in T cells. CD39 regulated the expression of IL-24, which sustained the expression of CD223 and IL-10 and maintained the cell viability. In summary, the generation of Treg-of-B (P) cells by Peyer’s patch B cells was through the CD200R-STAT6-CD39-IL-24 axis pathway.

**Supplementary Information:**

The online version contains supplementary material available at 10.1007/s00018-024-05268-2.

## Introduction

CD200 is a type I membrane protein that is expressed on a variety of cell types in the central nervous system, including B cells, T cells and neurons [1–3]. Its short cytoplasmic tail lacks docking domains and signal motifs, but it activates cells harboring its receptor, CD200R. Notably, CD200-deficient mice exhibit delayed resolution of lung inflammation due to heightened macrophage activity and increased susceptibility to influenza infection [4]. Furthermore, a low level of CD200 has been implicated in autoimmune diseases, while recombinant CD200 has shown potential for alleviating airway hyperresponsiveness in a murine model [5]. Moreover, CD200R activation could result in tumor progression, and silencing CD200 with an antibody or siRNA could enhance T-cell function in patients with Chronic lymphocytic leukemia, CLL [6]. Studies have highlighted the role of CD200 in augmenting CD4 + CD25 + regulatory T (Treg) cells through the induction of tolerogenic dendritic cells (DCs) [3, 7]. Researches have established the correlation between CD200 and CD200R in regulating immune responses and promoting regulatory T cell function [7–10]. In addition, CD200R activation can promote the differentiation of regulatory T cells by upregulating IL-10, IDO (indoleamine 2,3-dioxygenase) or Foxp3 [11]. However, the influence of CD200 on the differentiation of naïve T cells into Treg cells has not been determined. Notably, our previous microarray analysis data indicated that Treg-of-B (P) cells increase the expression of CD200 [12]. Based on these findings, we hypothesized that the interaction between CD200 and CD200R might play a role in the generation and function of Treg-of-B (P) cells.

CD39 plays an important role in regulating immune tolerance. In mice, CD39 is expressed on Treg cells. The absence of CD39 in mice leads to impaired immune regulatory function, as CD39-deficient regulatory T cells are unable to suppress the proliferation of CD4 + CD25- responder T cells [13]. Human Foxp3 + CD25^hi^ Treg cells express CD39, and CD39 expression is decreased in multiple sclerosis (MS) patients [14]. Furthermore, CD39 + regulatory T cells are generated in conditions such as rheumatoid arthritis, viral infection and the tumor microenvironment and play a role in modulating immune responses [15–17]. These CD39 + Treg cells efficiently suppress effector T cells by reducing cell proliferation and cytokine production, acting as suppressors of immune function.

IL-24 is a member of the IL-10 family and the IL-20 subfamily. In IL-4 transgenic mice with spontaneous inflammatory skin lesions, IL-24 expression is one hundredfold greater in mice with skin lesions than in wild-type mice [18]. This increase is likely attributed to IL-24 being one of the genes most strongly activated by STAT6 in Th2 cells [19]. In collagen-induced arthritis (CIA), IL-24 exerts inflammatory effects. The efficacy of soluble IL-20RB, which binds to IL-19, IL-20 and IL-24, in ameliorating CIA severity was comparable to that of soluble TNFR [20]. IL-20RB deficiency leads to severe contact hypersensitivity in mice, suggesting the suppressive role of IL-20RB [21]. IL-24 exerts anti-inflammatory effects in a variety of autoimmune diseases, including inflammatory bowel disease (IBD) and experimental autoimmune uveitis (EAU), by activating SOCS3 [22, 23]. 

In our study, we discovered that CD200, which is produced by Peyer’s patch B cells, plays a key role in initiating Treg-of-B (P) cell induction. The interaction between CD200 and CD200R upregulates CD39 expression by phosphorylating STAT6, which subsequently increases the expression of IL-24. IL-24 secreted by Treg-of-B (P) cells can modulate CD223, IL-10 production and the viability through paracrine or autocrine signaling. We demonstrated the significance of the CD200R-STAT6-CD39-IL-24 axis in the generation of Treg-of-B (P) cells.

## Materials and methods

Materials and methods are provided in the online supplementary information.

## Results

### Peyer’s patch B cells produced CD200 to induce STAT6 phosphorylation and CD200R expression on T cells

**Fig. 1:**
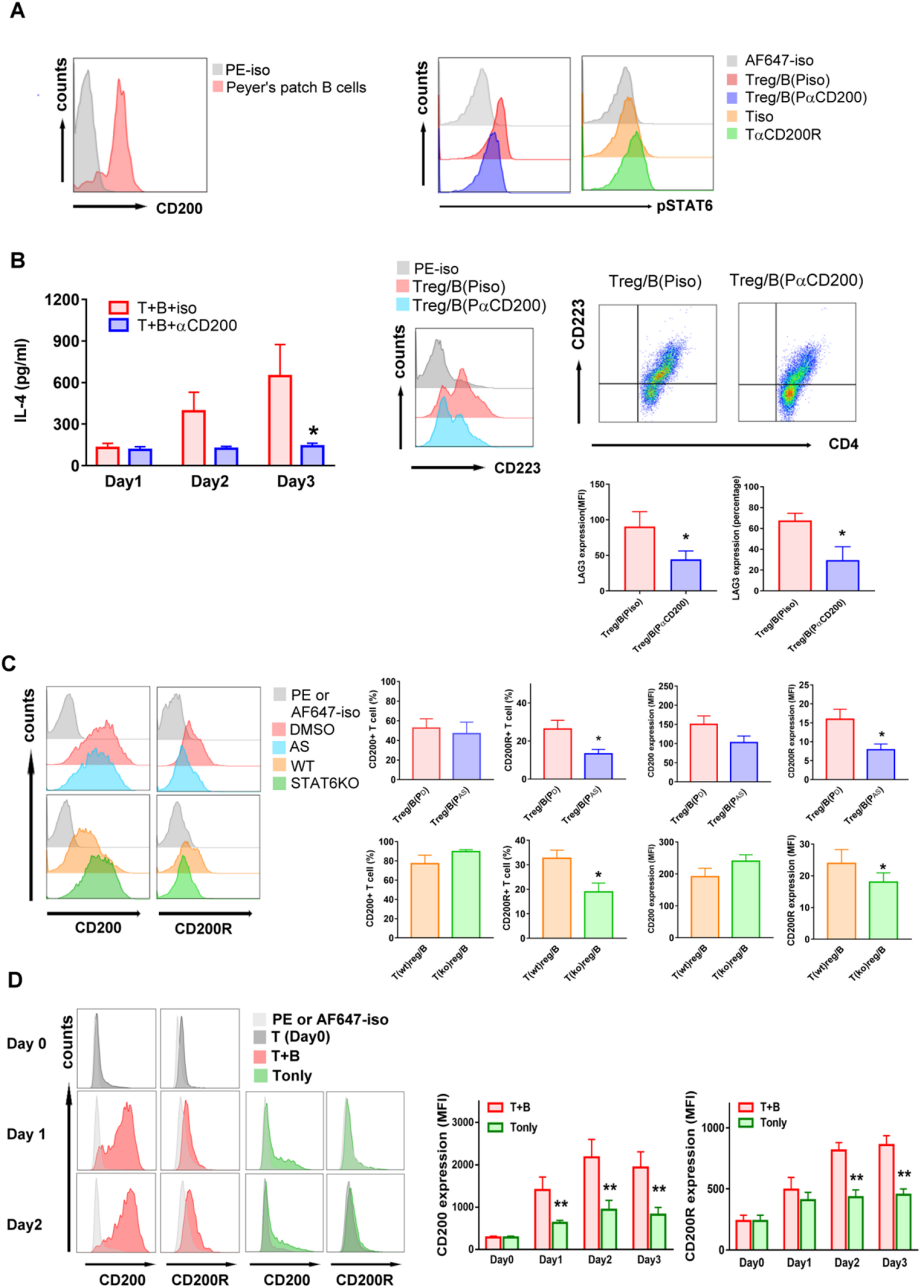
Peyer’s patch B cells provided CD200 to phosphorylate STAT6 in T cells, which in turn stimulate T cell expressing CD200R. (**A**, **B**) Flow cytometry analysis of CD200 expression by Peyer’s patch B cells, phosphorylated STAT6 and CD223. Naïve CD4 T cells cultured with Peyer’s patch B cells plus anti-CD3 and anti-CD28 antibodies in presence of anti-CD200 antibody (antagonist, red: isotype, blue: anti-CD200). Naïve CD4 T cells were stimulated with anti-CD3 and anti-CD28 antibodies in presence of anti-CD200R antibody (agonist, orange: isotype, green: anti-CD200R). The amount of IL-4 in culture supernatant was applied for ELISA analysis. (**C**) FACS analysis of the expression of CD200 and CD200R by Treg-of-B cell with phosphorylated STAT6 (DMSO and WT group) or without phosphorylated STAT6 (AS, AS1517499 and STAT6KO group). (red: DMSO group; orange: wild type group, WT; blue: AS group; green: STAT6KO group). (**D**) FACS analysis of the expression of CD200 and CD200R by T cells stimulated with anti-CD3 and anti-CD28 antibody with or without Peyer’s patch B cells for three days (green: T only group; red: T cultured with B, T + B, group). Data are representative of three to four different experiments. Results are expressed as the mean ± SEM. *p < 0.05, **p < 0.01 compared with isotype group, wild type group, DMSO group or T + B group

We demonstrated that Peyer’s patch B cells induced the generation of regulatory T cells by phosphorylating STAT6 [24]. In addition, our microarray data indicated that the expression of CD200 on Treg-of-B (P) cells is upregulated [12]. Previous studies have demonstrated that STAT6 is the downstream effector of CD200R in microglia [25]. Based on these findings, we hypothesized that Peyer’s patch B cells convert naïve T cells into regulatory T cells through the activation of CD200R. Our data demonstrated that Peyer’s patch B cells express CD200 (Fig. 1A. left). To confirm that the interaction between CD200 and CD200R can phosphorylate STAT6, we applied an anti-CD200 antibody during the Treg-of-B (P) cell generation step. The phosphorylation of STAT6 decreased when the CD200-CD200R interaction was interrupted, resulting in the decreased expression of IL-4 and CD223. (Fig. 1A, red and blue histogram, and 1B). In contrast, activation of CD200R on naïve T cells with an anti-CD200R antibody resulted in STAT6 phosphorylation (Fig. 1A, orange and green histograms). These data suggested that the CD200-CD200R interaction phosphorylates STAT6. To confirm this point of view, AS1517499 (labeled AS), a STAT6 inhibitor, and STAT6 knockout (STAT6KO) T cells were used in the Treg-of-B (P) cell generation system. While CD200 expression was unaffected by STAT6 phosphorylation, the CD200R level was decreased in the absence of STAT6 phosphorylation (Fig. 1C). We also demonstrated that without Peyer’s patch B cells, the expression of CD200 and CD200R in T cells was decreased (Fig. 1D). These findings suggested that Peyer’s patch B cells produced CD200 to activate CD200R, leading to STAT6 phosphorylation and increased expression of CD200R.

### The CD200-CD200R interaction regulates Treg-of-B (P) cell generation but does not contribute to the suppressive effects of Treg-of-B (P) cells

**Fig. 2:**
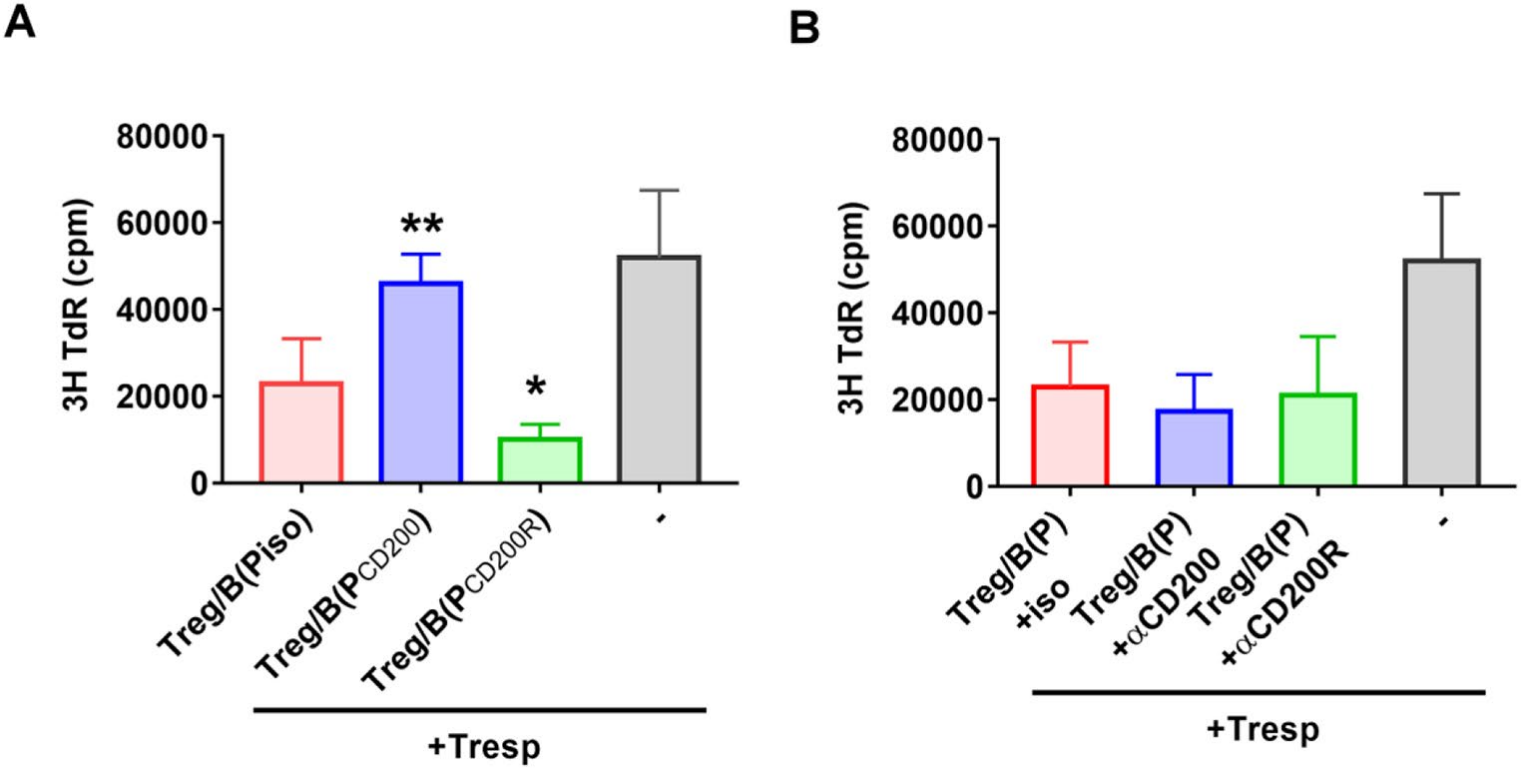
CD200-CD200R interaction participated in Treg-of-B (P) cell generation. To investigate whether CD200-CD200R interaction affects the generation of Treg-of-B (P) cells or act as the suppressive molecule, the agonist anti-CD200R antibody and antagonist anti-CD200 antibody were applied during Treg-of-B (P) cell generation or suppressive function assay. The functionality of Treg-of-B (P) cells was assessed based on their ability to inhibit the proliferation of responder T cells. Briefly, anti-CD200 (labeled as P_CD200_) or anti-CD200R (labeled as P_CD200R_) antibodies were administrated in T cell cocultured with Peyer’s patch B cells (Treg-of-B induction). Isotype antibody was used as control group (labeled as P_iso_). After three-day coculture, the three different groups of Treg-of-B (P) cells were harvested and cultured with CD4 + CD25- responder T cells (**A**). To investigate the role of CD200 in suppressive ability, Treg-of-B (P) cells were harvested and then cultured with responder T cells in presence of anti-CD200 (labeled as αCD200) or anti-CD200R (labeled as αCD200R) antibodies (**B**). To measure the responder T cell proliferative response, 1 µCi of 3 H-thymidine was added to the culture for the last 16 h. Thymidine uptake was determined using a β-counter. Data are representative of three different experiments. Results are expressed as the mean ± SEM. *p < 0.05, **p < 0.01 compared with isotype group

CD200 has the ability to suppress the response of CD200R-bearing cells and promote immune tolerance [1, 4, 8]. Considering that Treg-of-B (P) cells express CD200, we hypothesize that the CD200-CD200R interaction might participate in the induction and suppressive function of Treg-of-B (P) cells. To elucidate the role of CD200 in Treg-of-B (P) cell generation, a neutralizing antibody, an anti-CD200 antibody, and an agonist antibody, an anti-CD200R antibody, were administered to Peyer’s patch B cell-naïve T cells coculture systems. The results showed that interruption of the CD200-CD200R interaction reversed the suppressive effect of Treg-of-B (P) cells (Fig. 2A, blue bar), while activation of CD200R enhanced this suppressive effect (Fig. 2A, green bar). These results suggested that the CD200-CD200R interaction regulated the generation of Treg-of-B (P) cells. Furthermore, our study demonstrated that CD200 did not contribute to the suppressive function of Treg-of-B (P) cells. To test this hypothesis, naive T cells cultured with Peyer’s patch B cells were harvested and subjected to a suppressive function test in the presence of anti-CD200 or anti-CD200R antibodies. Figure 2B showed the ability of Treg-of-B (P) cells to inhibit the proliferation of responder T cells was independent of CD200 (Fig. 2B).

### Treg-of-B (P) cells expressed CD200 and CD200R to maintain the viability

**Fig. 3:**
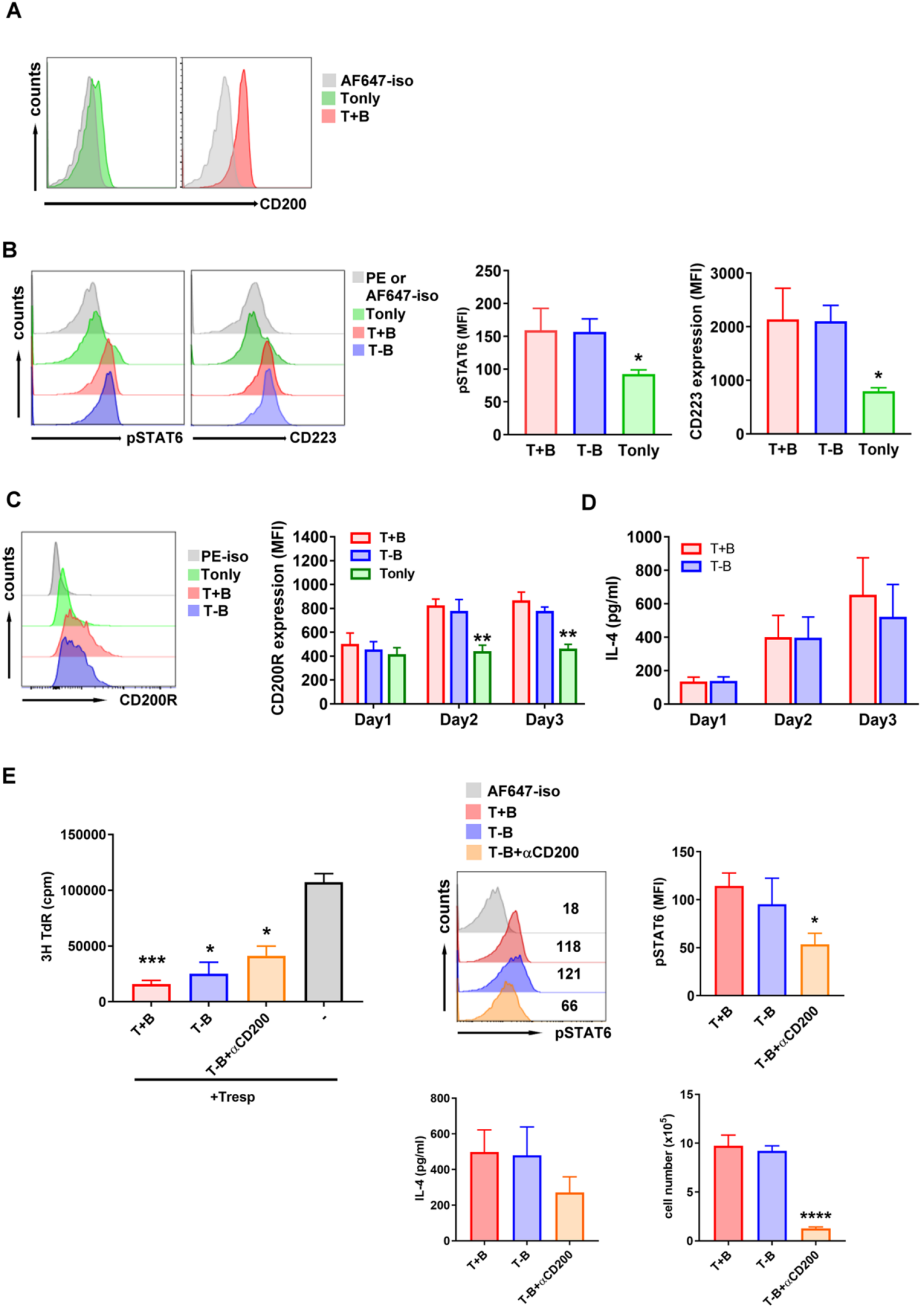
CD200-CD200R interaction between T cells helps T cells survive. (**A**) T cells cultured with Peyer’s patch B cells (labeled as T + B) could express CD200. (**B**, **C**) FACS analysis of the expression of phosphorylated STAT6, CD223 and CD200R. To determine the role of CD200 expressed by T cells, Peyer’s patch B cells were depleted after culturing with T cells one day. The remaining T cells were cultured for another two days (labeled as T-B). The culture supernatant was harvested for determination of the amount of IL-4 by ELISA (**D**). (**E**) To confirm that CD200-CD200R interaction between neighboring T cells was important for Treg-of-B (P) cell suppressive ability, anti-CD200 antibody was applied in the T-B groups (labeled as T-B + αCD200). These Treg-of-B (P) cells were harvested for suppression function test (left). Phosphorylated STAT6, the amounts of IL-4 and cell viability were determined by FACS analysis and ELISA, respectively (right). Data are representative of three to four different experiments. Results are expressed as the mean ± SEM. *p < 0.05, **p < 0.01, ***p < 0.005, ****p < 0.001, compared with T-B group or responder T cell only group (labeled as -)

We demonstrated that Peyer’s patch B cells promoted the expression of CD200 (Fig. 3A) and CD200R on T cells and that the interaction of CD200 and CD200R is important for Treg-of-B (P) cell generation (Fig. 2A). In the 3-day B-T-cell coculture, it was unclear whether Peyer’s patch B cells initially produced CD200 to activate naïve T cells, which were then sustained by their own CD200 expression, or if Peyer’s patch B cells produced CD200 throughout Treg-of-B (P) cell induction. To address this question, we designed a protocol in which B cells were depleted after one day of B and T coculture (labeled T-B), and the remaining T cells were cultured for an additional two days.

The results demonstrated that phosphorylated STAT6 and CD223, CD200R and IL-4, which are regulated by STAT6, exhibited similar expression patterns between the T and B cocultured groups and the B-cell depletion group (Fig. 3B-D, labeled T + B and T-B, respectively). T cells without an activating signal from Peyer’s patch B cells (labeled Tonly) exhibited lower levels of phosphorylated STAT6, CD200R and CD223. These results indicated that Peyer’s patch B cells provided the initial signal, CD200, to stimulate T cells and facilitate the expression of CD200 and CD200R by T cells. T cells expressing these two molecules can interact with neighboring T cells through the CD200-CD200R interaction, enhancing their regulatory function. To investigate the role of CD200 in Treg-of-B (P) cell generation, we applied a neutralizing anti-CD200 antibody to the T-B group. After Peyer’s patch B cells were depleted, the remaining T cells were cultured for an additional two days in the presence of the anti-CD200 antibody (labeled T-B + αCD200). To our surprise, even without continuous CD200R activation, Treg-of-B (P) cells still exhibited suppressive effects (Fig. 3E, T-B + αCD200 group). Further analysis revealed that disruption of the CD200-CD200R interaction resulted in decreased levels of phosphorylated STAT6 and IL-4, which led to decreased cell viability (Fig. 3E, right).

### CD39 expression on Treg-of-B (P) cells is regulated by STAT6 phosphorylation

**Fig. 4:**
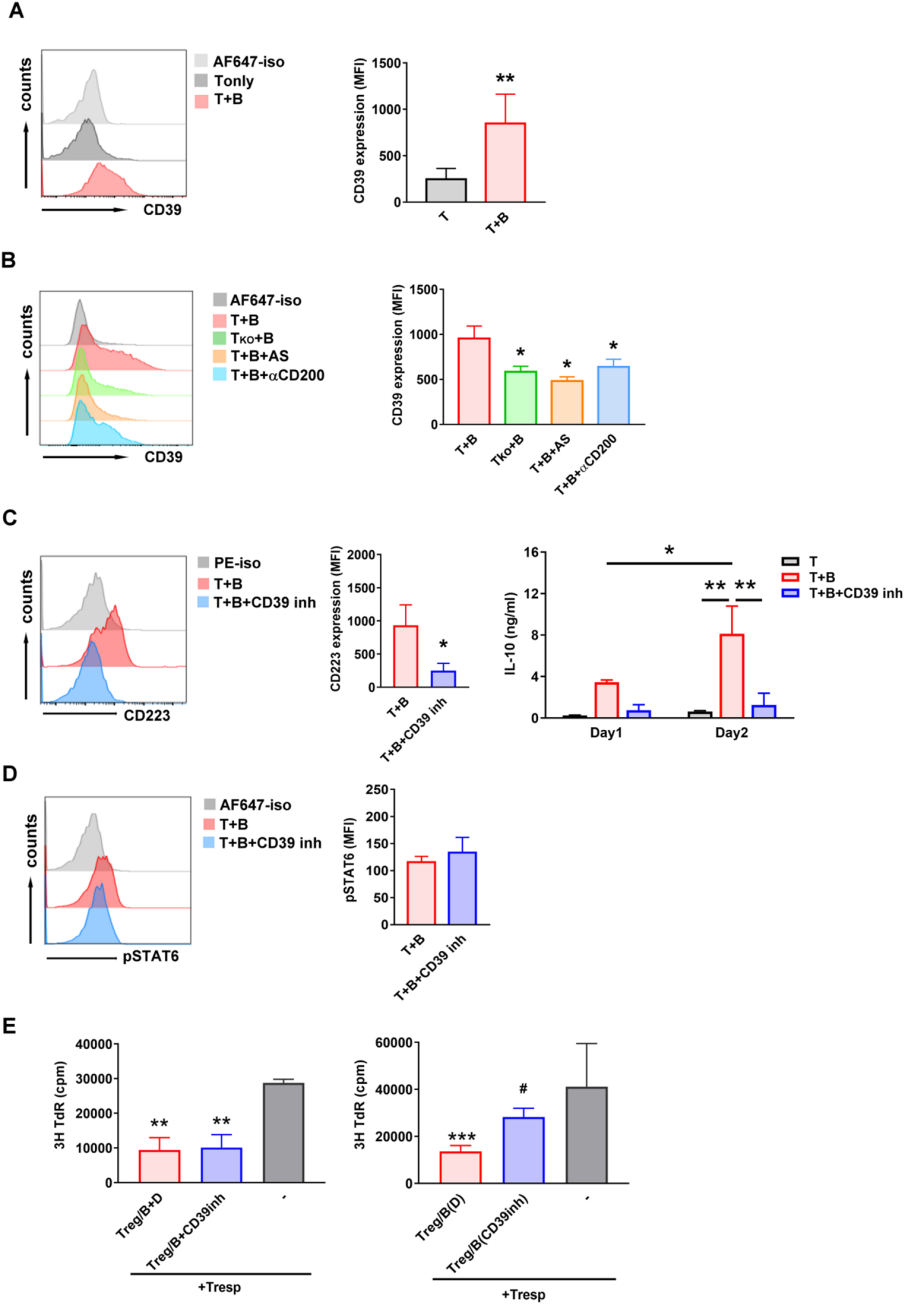
CD39, which is regulated by CD200-CD200R interaction, participated in Treg-of-B (P) cell generation. (**A**, **B**) FACS analysis of CD39 expression by wild type T cells (labeled as T + B), STAT6 knock T cells (labeled as T_ko_+B), T cells with STAT6 inhibitor AS (labeled as T + B + AS) or T cells with anti-CD200 antibody (labeled as T + B + αCD200), cultured with Peyer’s patch B cells for two days. (**C, D**) FACS analysis of CD223 and phosphorylated STAT6 by T cells cultured with Peyer’s patch B cells with (labeled as T + B + 39inh) or without (labeled as T + B) CD39 inhibitor for three days. Treg-of-B (P) cells were harvested and separated to two parts. One part for FACS analysis. The second part for detection of IL-10. After restimulation of Treg-of-B (P) cells, supernatant was harvested for ELISA. (**E**) To investigate the role of CD39 in Treg-of-B (P) cell suppressive ability, CD39 inhibitor was applied in the process of Treg-of-B (P) cell suppression function test (left). In addition, the role of CD39 in Treg-of-B (P) cell generation was evaluated. CD39 inhibitor was added in T cell cultured with Peyer’s patch B cells (right). Data are representative of three to four different experiments. Results are expressed as the mean ± SEM. *p < 0.05, **p < 0.01, ***p < 0.005, compared with T + B group or responder T cell only group (labeled as -). #*p* < 0.05, compared with Treg-of-B (P) DMSO group (labeled as Treg/B(D))

We demonstrated that Peyer’s patch B cells provided the initial activating signal (CD200) to activate naïve T cells via STAT6 phosphorylation and generated Treg-of-B (P) cells. STAT6 activation resulted in increased expression of CD200 and CD200R on Treg-of-B (P) cells, which plays a role in maintaining cell survival. However, CD200-CD200R engagement did not affect the suppressive ability of Treg-of-B (P) cells (Fig. 3E). Therefore, identifying the factor that is controlled by phosphorylated STAT6 and responsible for the suppressive effect of Treg-of-B (P) cells is necessary. Notably, CD39 has been mentioned as a marker for assessing the quality of regulatory T cells. These findings prompted us to investigate the expression of CD39 on Treg-of-B (P) cells. Our data suggested that Peyer’s patch B cells promoted CD39 expression on T cells, which was regulated by CD200 and STAT6 phosphorylation. In absence of phosphorylated STAT6, including using STAT6-deficient T cells or inhibition of phosphorylated STAT6 by inhibitor, Treg-of-B (P) cells decreased CD39 expression, suggested that CD39 expression was regulated by STAT6 (Fig. 4A, B). Inhibition of CD39 reduced CD223 expression and the production of IL-10, which were found to participate in the suppressive effect of Treg-of-B (P) cells (Fig. 4C). Conversely, blockade of CD39 had no effect on STAT6 phosphorylation, indicating that STAT6 regulated CD39 expression, but CD39 did not affect STAT6 expression (Fig. 4D). Previous studies have shown that CD39 + regulatory T cells exhibit suppressive effects by hydrolyzing extracellular ATP [14, 26]. In this study, we aimed to elucidate whether CD39 contributed to Treg-of-B (P) cell induction or served as a mediator of Treg-of-B (P) cell suppressive ability. Treg-of-B (P) cells were able to suppress the proliferation of responder T cells even in the presence of a CD39 inhibitor or A2AR inhibitor (Fig. 4E, left and Supplementary Fig. 1A), suggesting that CD39 does not participate in the suppressive effect of Treg-of-B (P) cells. In contrast, administration of a CD39 inhibitor or A2AR inhibitor during Treg-of-B (P) cell generation impaired the production of Treg-of-B (P) cells, which resulted in decreased suppressive activity (Fig. 4E right and Supplementary Fig. 1B).

### IL-24, which is controlled by CD39, regulates the expression of IL-10 and CD223 and maintains the viability of Treg-of-B (P) cells

**Fig. 5:**
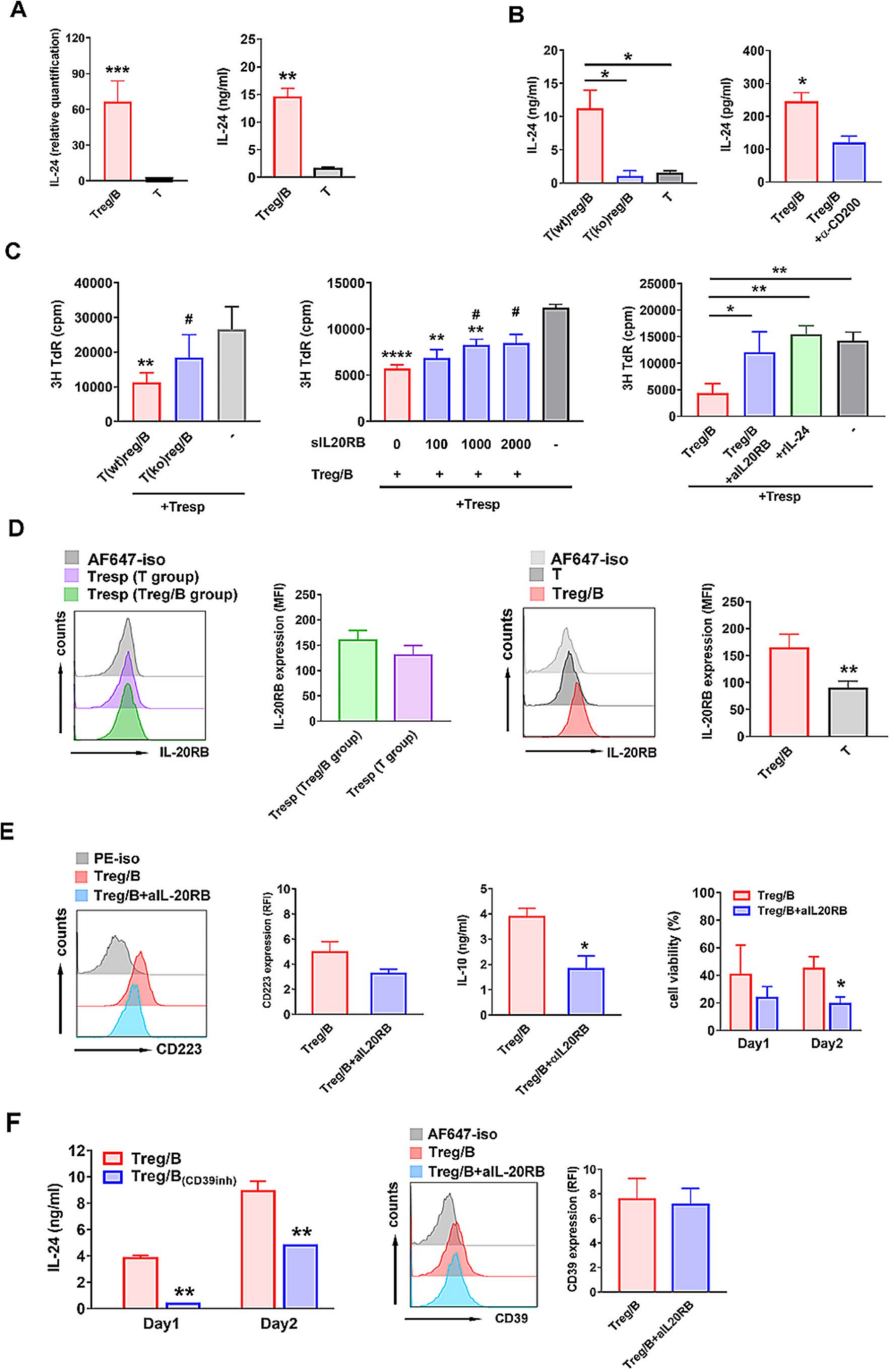
IL-24, which is regulated by CD200-CD200R interaction, participated in Treg-of-B (P) cell generation. (**A, B**) The expression of IL-24 was analyzed by quantitative PCR (QPCR) and ELISA. Wild type T cells or STAT6 knockout T cells were cultured with Peyer’s patch B cells (labeled as Treg/B, T(ko)reg/B, respectively). T cells only were applied as control group (labeled as T). To determine the role of CD200 in regulating IL-24, T cells were cultured with Peyer’s patch B cells in presence of anti-CD200 antibody (labeled as Treg/B + aCD200). After three days, T cells and Treg-of-B (P) cells were harvested for QPCR or restimulation for ELISA. (**C**) To determine the role of IL-24 in Treg-of-B (P) cell suppressive ability, wild type and IL-24 knockout T cells were used in Treg-of-B (P) cell generation (labeled as T(wt)reg/B and T(ko)reg/B, respectively). Wild type Treg-of-B (P) cells were harvested and applied for suppressive function test in presence of different concentration of soluble IL-20RB (0, 100, 1000 and 2000 pg/ml), or anti-IL20RB antibody. Responder T cells plus recombinant IL-24 (50 ng/ml) was used as control group. (**D**) FACS analysis of the expression of IL-20RB on T cells, Treg-of-B (P) cells and responder T cells. After three days generation, T cells and Treg-of-B (P) cells were harvested and cocultured with responder T cells. To distinguish responder T cells and T cells or Treg-of-B (P) cells, responder T cells were labeled with CFSE before coculturing. After two days coculture, four cell subsets were analyzed by FACS. Four cell subsets: Treg-of-B (P) cell (red color), T cell (dark gray), responder T cell (cultured with Treg-of-B (P) cell, green color), responder T cell (cultured with T cell, purple color). (**E**) To analyze whether IL-24 affect Treg-of-B (P) cell suppressive ability, analysis of the expression of CD223, the amount of IL-4 and cell viability were performed. Treg-of-B (P) cells were restimulated with or without anti-IL-20RB for two days. Cells were applied for analyzing CD223 expression and cell viability. Culture supernatant was harvested for analyzing IL-10 level. (**F**) (left) T cells were cultured with Peyer’s patch B cells with or without CD39 inhibitor (labeled as Treg/B and Treg/B_(CD39inh)_, respectively). After three days, different Treg-of-B (P) cell groups were applied for restimulation and culture supernatant was harvested for analysis of IL-24 production. (right) Treg-of-B (P) cells were restimulation with or without anti-IL-20RB. After two days, cells were harvested for analysis of CD39 expression. Results are expressed as the mean ± SEM. *p < 0.05, **p < 0.01, ***p < 0.005, ****p < 0.001 compared with T group, Treg/B group, Treg/B + αCD200 group or responder T cell only group (labeled as -). ^#^*p* < 0.05, ^##^*p* < 0.01, compared with T(wt)reg/B group, T(ko)reg/B group or sIL-20RB (0) group

Our data suggested that Treg-of-B (P) cells increased gene and protein expression of IL-24 compared to that of CD25- T cells (Fig. 5A). Consistent with the findings of previous studies, our results showed that IL-24 production was regulated by STAT6. Treg-of-B (P) cells lacking STAT6 were unable to secrete IL-24 (Fig. 5B, left). Our data suggested that the interaction between CD200 and CD200R served as the initial signal for the phosphorylation of STAT6. Disruption of CD200-CD200R engagement by an anti-CD200 antibody reduced IL-24 production by Treg-of-B (P) cells, highlighting the involvement of the CD200-STAT6-IL-24 axis (Fig. 5B, right). To explore the involvement of IL-24 in the function of Treg-of-B (P) cells, which are known to regulate immune responses [21, 23], we generated Treg-of-B (P) cells from IL-24-deficient T cells. The results demonstrated that the absence of IL-24 decreased the ability of Treg-of-B (P) cells to inhibit the proliferation of responder T cells. Moreover, blocking IL-24 with soluble IL-20RB and an anti-IL-20RB antibody impaired the suppressive function of Treg-of-B (P) cells (Fig. 5C). Surprisingly, recombinant IL-24 alone did not inhibit the proliferation of responder T cells (Fig. 5C and Supplementary Fig. 2). This finding suggested that Treg-of-B (P) cells could upregulate the expression of IL-20RB by responder T cells and increase the sensitivity of responder T cells to the IL-24 signal. To investigate this possibility, responder T cells were cultured with either Treg-of-B (P) cells or activated T cells, and IL-20RB expression was analyzed via FACS analysis. No significant difference in the expression of IL-20RB on responder T cells was detected after culture with either Treg-of-B (P) cells or activated T cells (Fig. 5D, left), suggesting that the effect of IL-24 was not direct on responder T cells. In contrast, the expression of IL-20RB on Treg-of-B (P) cells was greater than that on activated T cells (Fig. 5D, right). These findings suggested that IL-24 might regulate the function of Treg-of-B (P) cells. Inhibiting the IL-24 signal with an anti-IL-20RB antibody decreased CD223 expression, IL-10 production and cell viability (Fig. 5E). These findings suggested that IL-24 plays a role in the suppressive function of Treg-of-B (P) cells. Moreover, we determined the mechanisms underlying Treg-of-B (P) cell generation. Disrupting CD39 activation led to decreased IL-24 production, whereas blocking IL-24 stimulation did not affect the expression of CD39 (Fig. 5F). These data suggested that IL-24 was regulated by CD39 (Fig. 5F).

**Fig. 6:**
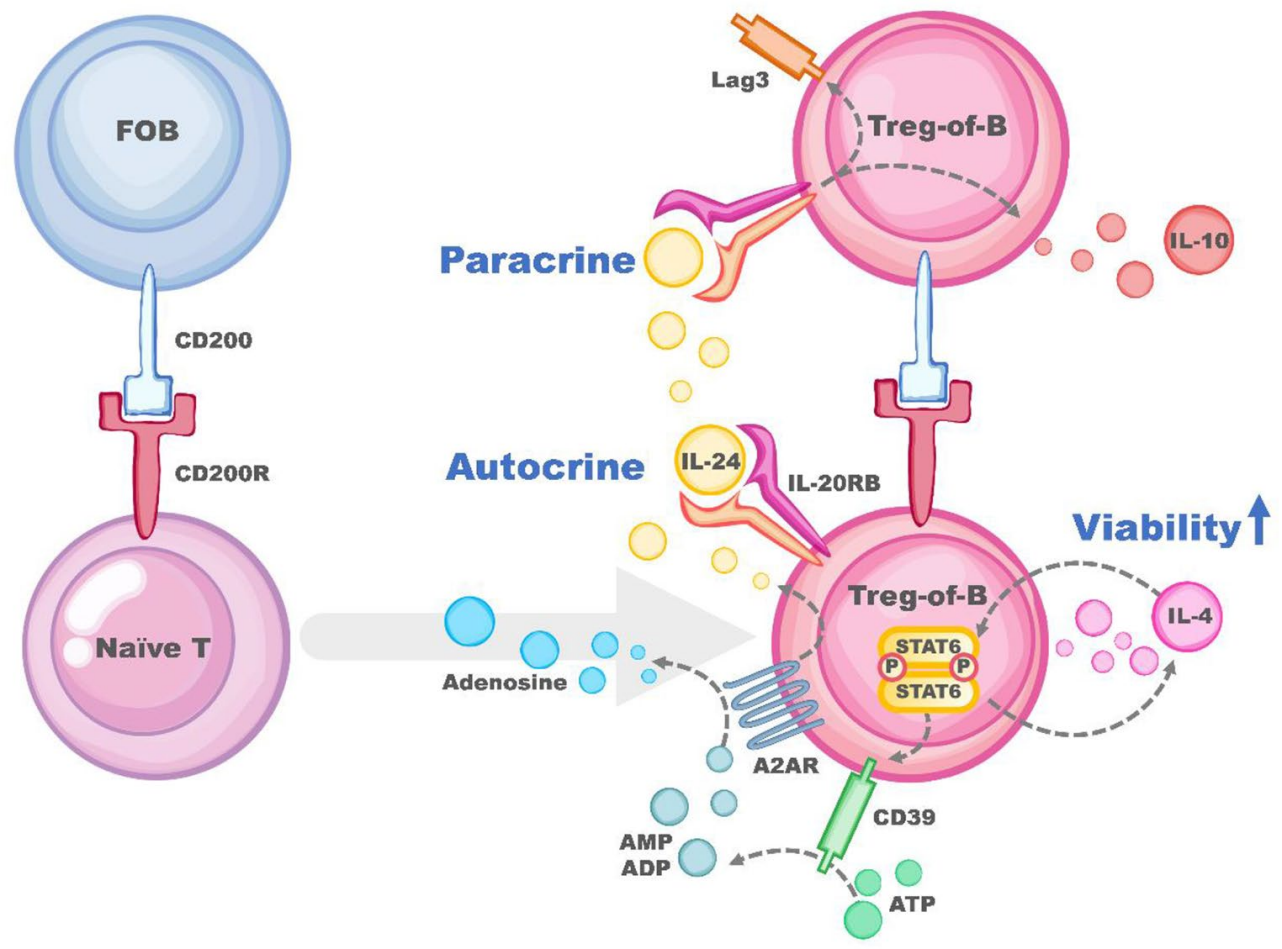
Peyer’s patch B cells induce Treg cells with CD200R-STAT6-CD39-IL-24 axis pathway. Peyer’s patch B cells provided the first signal, CD200, to activate T cells, with the following CD39 expression. CD39 regulate the increased expression of IL-24 that could sustain the CD223 and IL-10 production, and upregulate Treg-of-B (P) cell viability

## Discussion

The role of B cells in tolerance induction has been extensively documented [27–33]. Our group has demonstrated that B cells can induce regulatory T cells via STAT6 phosphorylation [12, 24, 34–38]. Our objective was to explore how B cells induce Treg-of-B (P) cell generation. In this study, we demonstrated the importance of CD200, which is expressed by Peyer’s patch B cells, in generating Treg-of-B (P) cells. When the CD200 signal is abrogated, Treg-of-B (P) cell generation is suppressed. In addition, CD200R activation on T cells phosphorylates STAT6, and, in turn, phosphorylated STAT6 promotes CD200R expression (Fig. 1C). These data suggested that B cells induced the expression of CD200 on T cells, establishing a positive feedback loop between CD200 and CD200R. This feedback loop helps to maintain STAT6 phosphorylation and promotes the survival of Treg-of-B (P) cells (Fig. 3). Many studies have demonstrated the role of CD200 in immune responses. The interaction of CD200-CD200R inhibits the function of M1-like macrophages. CD200-deficient mice exhibit an enhanced microglial response and an earlier onset of EAE [1]. Moreover, CD200 suppresses NK cell cytolytic activity and basophil activation [39, 40]. CD200 also regulates T cells via direct or indirect mechanisms. In the indirect route, CD200 activates CD200R-expressing DCs, which in turn induces the generation of regulatory T cells [3, 7]. In the direct route, CD200 expressed by DCs can directly activate CD200R on T cells. The CD200-CD200R interaction leads to a shift in cytokine production from Th1 to Th2 and promotes the induction of regulatory T cells [7, 9, 41]. Gorczynski et al. demonstrated that CD200 increased graft survival in patients that underwent heart and skin transplantation. Two weeks after transplantation, the graft survived despite decreased CD200 expression. Conversely, blocking CD200 at the beginning of the graft transplantation resulted in graft loss. These findings suggested that CD200 participated in the development of tolerance but not its maintenance [8]. Our data demonstrated that CD200, which is produced by Peyer’s patch B cells, serves as the initial signal for initiating the induction of Treg-of-B (P) cells. Our previous studies revealed two major B-cell subsets, follicular B (FOB) cells and CD23^lo^ CD21^lo^ B cells. Among these subsets, FOB cells display a better ability to induce Treg-of-B (P) cells [12]. We assessed the gene expression of CD200 in these two cell subsets and found that CD200 levels were higher in the FOB cell group (data not shown). Engagement of CD200 and CD200R expressed by CD4 + CD25- T cells induces STAT6 phosphorylation, which is a crucial step in the generation of Treg-of-B (P) cells [24]. Stimulation of naïve T cells with anti-CD3 and anti-CD28 antibodies did not generate regulatory T cells. The interaction between CD200 and CD200R is critical for the generation of Treg-of-B (P) cells, as evidenced by the lack of Treg-of-B (P) cell generation when the CD200-CD200R interaction was disrupted by an anti-CD200 antibody (Fig. 2A).

Given that T cells express both CD200 and CD200R, T cells are likely to be continuously activation by interactions with neighboring T cells. To test this hypothesis, we initially cultured T cells and B cells together for one day and subsequently depleted the B cells. Surprisingly, even in the absence of Peyer’s patch B cells for the following two days, the remaining T cells exhibited consistent CD200R activation, as well as increased expression of phosphorylated STAT6, CD200R, CD223 and IL-4 (Fig. 3B-D, T-B group). When the anti-CD200 antibody was applied to the T-B cells, the activation of CD200R was interrupted (Fig. 3E, T-B + a-CD200 group), suggesting that the interaction between T cells occurred through the CD200-CD200R interaction. In addition to the phosphorylated STAT6 and IL-4 production, we found that the interaction of CD200-CD200R in T cells maintained the viability of Treg-of-B (P) cells. This might explained the suppressive ability of T-B + aCD200 group Treg-of-B (P) cell partially decreased, compared to T + B group (Fig. 3E). In our Treg-of-B (P) cell generating system, after depleting Peyer’s patch B cells, total CD4 + T cells were harvested for the following tests. The cell number of Treg-of-B (P) cells were recalculated and then cultured with responder T cells with 1:1 ratio. In order to reduce the differences between batches, LAG3 expression on wild type or isotype group Treg-of-B (P) cells was determined and over 90% LAG3 + Treg-of-B (P) cells in total T cells were accepted to do the suppressive test. In T-B + aCD200 group, the suppressive ability showed partially reversed. It is likely due to the decrease in Treg-of-B (P) cell numbers during the process of culturing with responder T cells.

A previous study revealed that macrophages can respond to changes in the environmental conditions through the activation of cAMP/CREB signaling via G protein-coupled receptor (GPCR) ligation, which in turn regulates CD39 expression [42, 43]. In addition, CD200 promoted macrophage polarization to the M2 phenotype through the cAMP/CREB signaling pathway [44]. Based on these findings, CD39 expression might be modulated by CD200. Our data demonstrated that in the absence of CD200 produced by Peyer’s patch B cells, both STAT6 phosphorylation and CD39 expression were decreased. Furthermore, inhibition of CD39 did not affect STAT6 phosphorylation, suggesting that CD39 expression is regulated by CD200. CD39 plays a role in Treg-of-B (P) cell generation. Without CD39 activation, Treg-of-B (P) cells produced lower levels of IL-10 and CD223, resulting in decreased suppressive ability. Previous reports have indicated that CD39 contributes to the suppressive activity of Foxp3 + regulatory T cells. It acts by hydrolyzing immunogenic ATP into AMP, thereby suppressing immune responses [14]. Contrary to previous findings, our data suggested that abolishing CD39 did not affect the suppressive ability of Treg-of-B (P) cells. The inhibition of responder T-cell proliferation by Treg-of-B (P) cells was adenosine-independent, as demonstrated in Fig. 4. Although IL-4 is known to inhibit CD39 expression via STAT6 phosphorylation, we found that the absence of STAT6 phosphorylation actually leads to a decrease in CD39 expression [45]. Further investigation is required to determine the detailed mechanism underlying this observation.

In our attempt to identify molecules regulated by phosphorylated STAT6, we discovered that IL-24 could be a potential candidate [19]. IL-24, similar to IL-19 and IL-20, belongs to the IL-10 superfamily due to structural similarities [46]. These three cytokines, IL-19, IL-20 and IL-24, share the same receptor subunit, IL-20RB. Wahl et al. demonstrated that IL-20RB-deficient mice exhibited upregulated T-cell responses following DNA vaccination or in a disease model of contact hypersensitivity. There was an increase in IFNγ-producing T cells, while the number of IL-10-producing T cells decreased in IL-20RB-deficient mice. Dendritic cells exhibit similar T-cell priming abilities in both wild-type and IL-20RB-deficient mice, suggesting that IL-24 indeed modulates immune responses by regulating T cells [21]. In our study, we observed higher expression of IL-24 in Treg-of-B (P) cells than in CD25 + regulatory T cells and CD25- T cells (Fig. 5). IL-24 plays an important role in the suppressive ability of Treg-of-B (P) cells. However, we demonstrated that the effect of IL-24 is not direct on responder T cells. Instead, IL-24 acts via autocrine signaling to promote CD223 and IL-10 expression on Treg-of-B (P) cells.

In summary, this study demonstrated that Peyer’s patch B cells provide the initial signal, CD200, to activate T cells. Subsequently, CD39 expression is induced, and increased IL-24 facilitates the production of CD223 and IL-10. These factors collectively enhance the viability of Treg-of-B (P) cells, as depicted in Fig. 6.

### Electronic supplementary material

Below is the link to the electronic supplementary material.

Supplementary Material 1

## Data Availability

All data generated or analyzed during this study are included in this published article and its supplementary information files.

## Abbreviations

CTL: Cytotoxic T lymphocyte
DCs: dendritic cells
CIA: collagen-induced arthritis
IBD: inflammatory bowel disease
Treg cell: regulatory T cells
MS: multiple sclerosis
GPCRs: G-protein-coupled receptors
