# Supplementary material for: CD200R activation on naïve T cells by B cells induces suppressive activity of T cells via IL-24
Source: Cell Mol Life Sci. 2024 May 23;81(1):231. doi: 10.1007/s00018-024-05268-2 (PMC11116298; doi:10.1007/s00018-024-05268-2)
Supplement: Supplementary file 1 — Supplementary Material 1 [file 18_2024_5268_MOESM1_ESM.docx]

Supplementary information

Title: CD200R Activation on Naïve T cells by B Cells Induces Suppressive Activity of T Cells via IL-24

Journal name: Cellular and Molecular Life Sciences

Authors: Kuan-Hua Chu and Bor-Luen Chiang

Correspondence to:

Dr. Bor-Luen Chiang

Department of Pediatircs

National Taiwan University Hospital

**Materials and Methods**

**Materials and Methods**

***Animals***

IL-24 knockout (IL-24KO) B6 mice and STAT6 knockout (STAT6KO) BALB/c background mice, were obtained from Jackson Laboratory (Bar Harbor, ME). C57BL/6 mice, BALB/c mice aged 6-8 weeks were obtained and maintained in the National Laboratory Animal Center. STAT6KO and IL-24KO mice were bred in the National Laboratory Animal Center (Taiwan). The animal study protocol was approved by the Institutional Animal Care and Use Committee (IACUC) of the College of Medicine at National Taiwan University.

***Preparation of Treg-of-B cells***

The protocol for Treg-of-B cell generation and the characteristics of these cells are as previously described.^24^ Naïve CD4 T cells from spleens (wild type, STAT6KO, or IL4KO) were enriched to a purity of over 90% using negative immunomagnetic depletion (EasySep, STEMCELL Technology, Canada). B cells from Peyer's patch were isolated via immunomagnetic positive selection (IMag, BD Pharmingen), resulting in a purity of 90-95% based on B220 expression. CD4+CD25- T cells were cultured with B cells (B:T=1:1) in the presence of soluble anti-CD3 and anti-CD28 (0.5 μg/ml) in RPMI-1640 culture medium supplemented with 5% FBS, 25 mM HEPES, 4 mM L-Gln, and antibiotics (100 U/ml penicillin, 100 μg/ml streptomycin, and 0.25 μg/ml amphotericin) for 3 days.

For determination of the role of CD200 in Treg-of-B (P) generation, 10 μg/ml antagonist antibody anti-CD200 (OX90, Biolegend, San Diego, CA) and agonist antibody anti-200R (OX110, Biolegend) were applied in B-T coculture. Identification of the role of CD39 in Treg-of-B (P) cell generation, the inhibitors, ARL67156, 50μM, (ATP analogs) and POM-1, 20μM, (enzymatic activity inhibitor) were used in B-T coculture. The A2AR antagonist, ZM241385, 10μM, was used to investigate the role of adenosine A2A receptor (A2AR). The role of IL-24 in Treg-of-B (P) generation was determined by applying anti-IL-20RB (10μg/ml) in B-T coculture.

***Suppressive function***

The assessment of suppressive function, which refers to the ability of Treg cells to inhibit the proliferation of responder T cells, has been previously described. ^24^ After three days of generating Treg-of-B (P) cells, they were harvested and co-cultured with CD25-CD4+ T cells (as responder T cells) and mitomycin c-treated splenocytes (antigen-presenting cells) at a concentration of 25 μg/ml in a 37°C environment for 30 minutes. The culture also stimulated with anti-CD3 and anti-CD28 at a concentration of 1 μg/ml for 96 hours. To measure the proliferative response, 1 μCi of 3H-thymidine was added to the culture for the last 16 hours. Thymidine uptake was determined using a β-counter (Packard Instrument Co., Meriden, CT, USA) and expressed as cpm (counts per minute). To determine the effects of molecules participating in Treg-of-B (P) cell suppressive ability, all antibodies, including anti-CD200, anti-200R and anti-IL-20RB antibody; inhibitors (ARL67156, and POM-1), antagonist, ZM241385, were added at the Treg-of-B (P) cell suppressive function test.

***Real-time quantitative-polymerase chain reaction (RT-QPCR)***

Total RNA was extracted following the manufacturer's instructions using TRIzol reagent (Invitrogen). First-strand cDNA was synthesized with random hexamers using Moloney murine leukaemia virus (MMLV) reverse transcriptase (Clontech). RT-QPCR was conducted using SYBR Green for quantitative PCR (Applied Biosystems) along with gene-specific primers. The analysis was performed using the 7500 FAST Real-Time PCR System (Applied Biosystems). GAPDH was used as an endogenous control. The results were normalized to GAPDH expression and analyzed using the ΔΔCt method. The relative multiples of changes in mRNA expression were determined by calculating 2^–ΔΔCt^. GAPDH forward: 5’-GATGGGTGTGAACCACGAGA-3’, reverse: 5’-AGATCCACGACGGACACAT-3’. IL-24 forward: 5’-AGCCTGCCCAACTTTTTGTG-3’, reverse: 5’- CCAGTGCTCCTGCTAGGGT-3’.

***Cytokine detection by ELISA***

IL-4, IL-10, and IL-24 production were assessed using an ELISA kit (R&D, Minneapolis, MN, USA) following the manufacturer's instructions.

***Fluorescence-activated cell sorting (FACS) analysis***

For cell surface marker staining, monoclonal antibody (mAb) against CD200 and LAG3 were purchased from BD Pharmingen; mAb against CD200R and CD39 were purchased from Biolegend (San Diego, CA, USA). Phosphorylated STAT (pSTAT) was stained with mAbs against pSTAT6 (BD Phosflow) followed by an intracellular staining protocol. For determination of IL-20RB expression on Treg-of-B (P) cells or responder T cells, responder T cells were labeled with CFSE 5μM on day one then cultured with Treg-of-B (P) cells for three days. On day three, all cells were harvested and stained with anti-IL-20RB, which was purchased from eBioscience (San Diego, CA, USA). Cell viability was determined by FVS780 (BD Pharmingen) staining. FVS780 negative was considered as viable cells. For determination of CD39 expression, Treg-of-B (P) cells were harvested and restimulated with or without anti-IL-20RB antibody for two days. CD39 expression was evaluated by FACS analysis. Cells were analyzed on a FACSCalibur and FACSLyric (BD Biosystems, Franklin Lakes, NJ, USA). Data were analyzed with FlowJo version 10.

***Statistical analysis***

The results were expressed as the mean±standard error of the mean (SEM). Statistical analyses were performed using GraphPad Prism VII software (GraphPad Software, La Jolla, CA). Two-group comparisons were made with Mann-Whitney test. Groups of three or more were analyzed by one-way ANOVA with a Kruskal-Wallis test. A *p* values < 0.05 were considered significant.

**Figures**


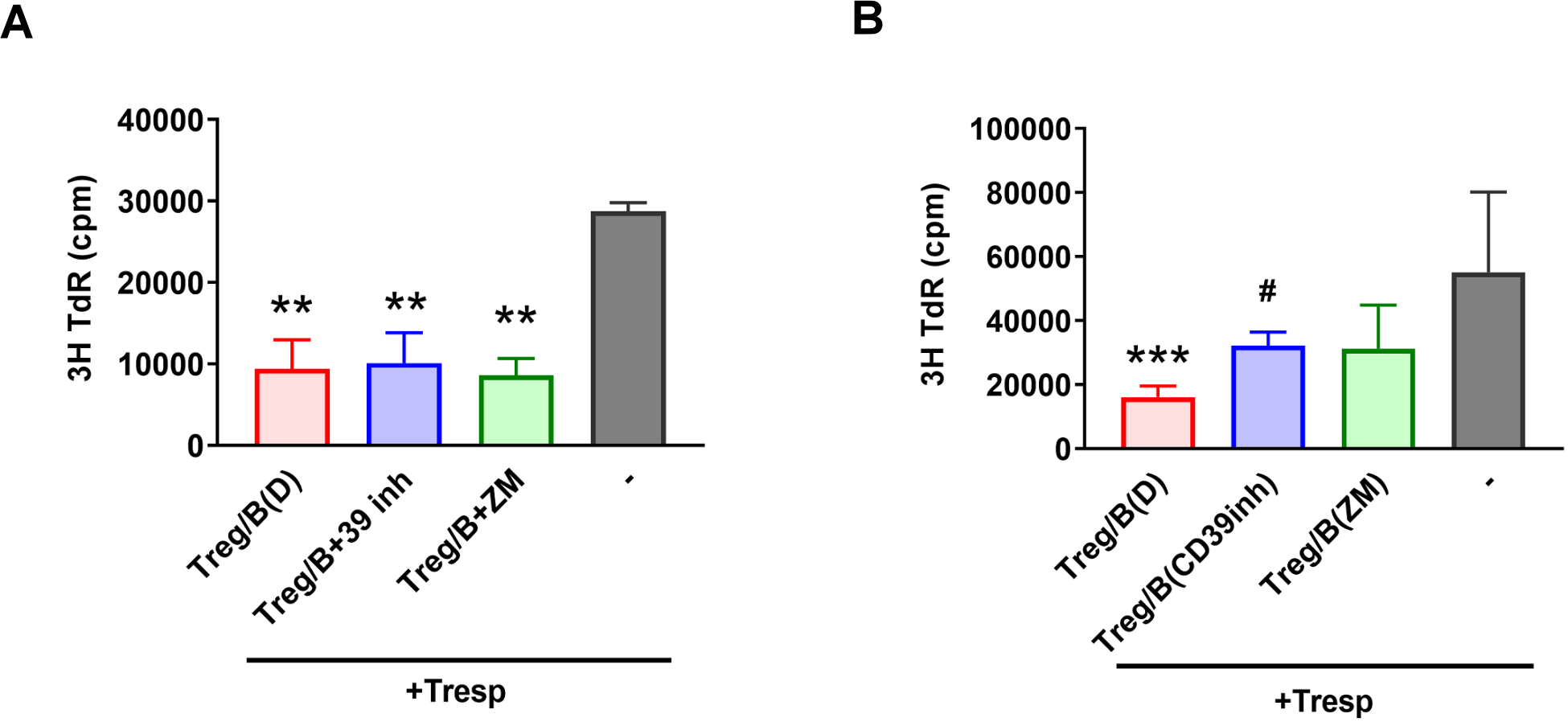


Supplementary Figure 1.

A.


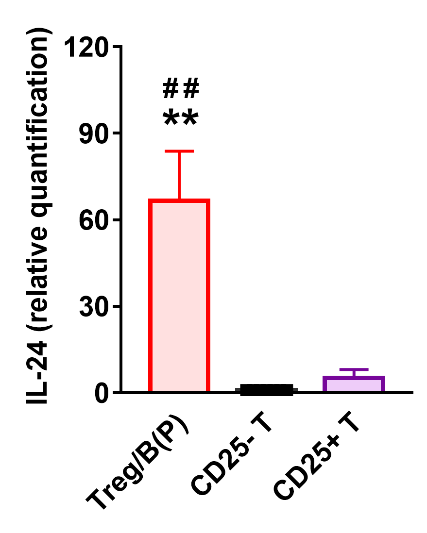


B.


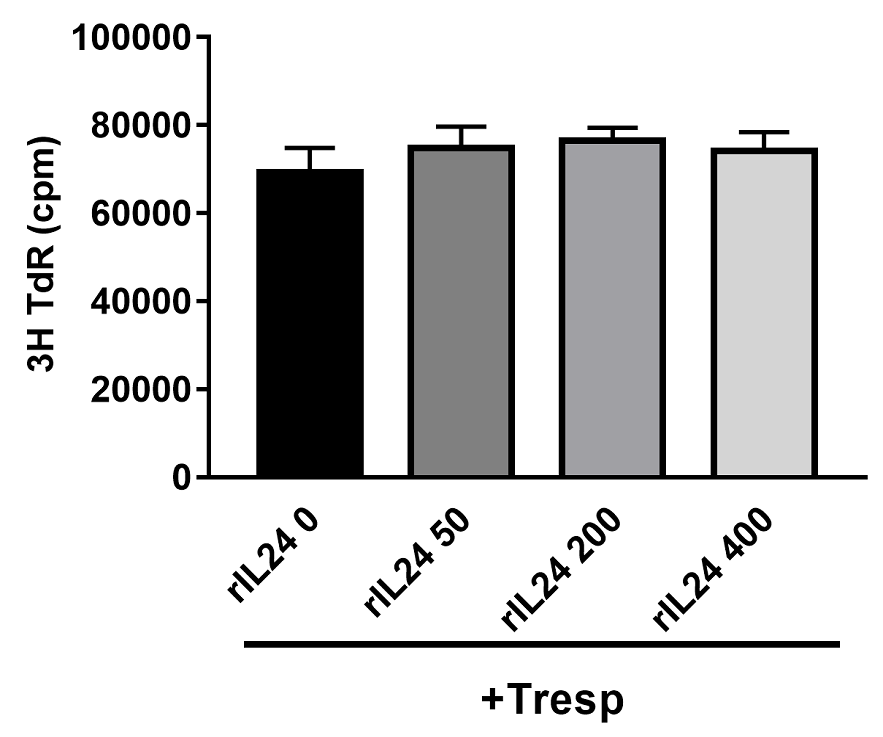


Supplementary Figure 2.

**Figure Legends**

**Supplementary Figure 1. CD39 participated in Treg-of-B (P) cell induction.** To identify the role of CD39 in Treg-of-B (P) cells, the CD39 inhibitor and A2AR inhibitor (labeled as ZM) were added in Treg-of-B (P) cell suppression functional test (A) or in the process of Treg-of-B (P) cell induction (B). The data showed that inhibition of CD39, or A2AR, the adenosine receptor, would reverse the suppression ability of Treg-of-B (P) cells, but not affect the generation of Treg-of-B (P) cells. Data are representative of three to four different experiments. Results are expressed as the mean±SEM. ***p*<0.01, ****p*<0.005, compared with T+B group or responder T cell only group (labeled as -). #*p*<0.05, compared with Treg-of-B (P) DMSO group (labeled as Treg/B(D))

**Supplementary Figure 2. IL-24 would not suppress responder T cell proliferation.** (A) The gene expression of IL-24 in Treg-of-B (P) cell, CD25- T cell and CD25+ tTreg cells were determined by real-time PCR. (B) Recombinant IL-24 (0, 50, 200 and 400 ng/ml) was administrated in responder T cells stimulated with anti-CD3 and anti-CD28 antibodies. Thymidine incorporation was evaluated for determination of the effect of IL-24. Data are representative of three different experiments. ***p*<0.01, compared with CD25- T cell, ##*p*<0.01, compared with CD25+ tTreg cell.
